# Findings From the Great British and Northern Ireland Botulinum Toxin Survey: Treatment Outcomes, Patient Experience, and Regulations From a Cross-Sectional Survey

**DOI:** 10.1093/asjof/ojaf115

**Published:** 2025-09-16

**Authors:** Lee Smith, José Francisco López-Gil, Julia Gawronska, Yvonne Barnett, Laurie Butler, Helen Keyes, Dong Keon Yon, Damiano Pizzol, Masoud Rahmati, Roshan Ravindran

## Abstract

**Background:**

Given rising demand for botulinum toxin (BoNT) treatments and limited data on safety, ethics, and regulation, a national survey explored experiences with cosmetic BoNT in the United Kingdom.

**Objectives:**

To conduct a national observational survey with the aim to capture real-world experiences of cosmetic BoNT in the United Kingdom.

**Methods:**

A cross-sectional online survey gathered data on experiences with cosmetic BoNT injections across the United Kingdom. Participation was open to adults (≥18 years) who had received cosmetic BoNT treatment.

**Results:**

A total of 919 participants completed the survey and were predominantly female, white, and had a high household income. Commonly reported acute complications were bruising/swelling (26.1%), and headache (24.7%). Commonly reported long-term complications were BoNT resistance (2.9%), social withdrawal (2.7%), nerve damage (2.5%), and dry eyes/vision problems (2.5%). In total, 66% stated their treatment was administered by a prescriber, 28% said it was not. Among those treated by a non-prescriber, 40% reported that a prescriber was present during the consultation, 42% said no prescriber was present, and 17% were unsure. Surprisingly, a few had not signed a consent form (8%), 11% were not informed of treatment risks, and 18% were not told how to respond to complications. A large majority expressed support for enhanced oversight, with 57.8% favoring significantly stricter regulation, and 31.3% somewhat stricter regulation.

**Conclusions:**

Cosmetic BoNT can offer high satisfaction and a favorable safety profile when administered appropriately. However, findings highlight key vulnerabilities: inconsistent practitioner qualifications, gaps in informed consent, insufficient complication support, and weak regulation.

**Level of Evidence:3 (Therapeutic):**

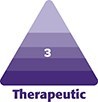

Botulinum toxin (BoNT) injection is among the most common cosmetic procedures worldwide, with millions of treatments administered annually.^1^ In the United Kingdom alone, an estimated 900,000 cosmetic BoNT injections are performed each year.^2^ The procedure's popularity is likely driven by its minimally invasive nature, effective wrinkle reduction, and reported high patient satisfaction rates.^3^ Specifically, patient satisfaction with BoNT type A for esthetic facial treatment has been reported to be between 65% to over 90% across clinical studies, though recent evidence suggests that satisfaction may vary by age group, treatment history, and product brand, with younger patients typically reporting higher satisfaction.^4,5^ Esthetic use of BoNT type A is supported by an extensive and continually expanding body of clinical literature. Multiple formulations, including onabotulinumtoxinA (Allergan, Irvine, CA), abobotulinumtoxinA (Galderma, Fort Worth, TX), incobotulinumtoxinA (Merz Pharmaceuticals, Frankfurt, Germany), and prabotulinumtoxinA (Evolus, Newport Beach, CA), have demonstrated efficacy and safety in a variety of cosmetic applications, particularly for dynamic facial rhytides.^6-9^ OnabotulinumtoxinA remains the most extensively studied formulation in both clinical and nonclinical publications since its introduction in the 1980s.^10^

These products form the cornerstone of modern minimally invasive facial rejuvenation. When used appropriately in suitable candidates, BoNT has been reported to have a strong safety profile, with predictable results and relatively few adverse effects.^11^ Serious systemic complications are likely rare—no long-term or life-threatening adverse effects have been documented for cosmetic BoNT in the scientific literature.^11^ Most treatment-emergent effects are mild and transient (eg, localized pain, bruising, and headache). Indeed, a recent meta-analysis of clinical trials found an overall complication rate of ∼16%, primarily consisting of minor issues like headache or skin reactions.^12^

Another important aspect of BoNT use is the psychosocial dimension. Patients seek cosmetic injections for appearance-related reasons that can be intertwined with mental well-being. Indeed, a subset of individuals may have underlying psychological conditions. For example, studies indicate that ∼10% to 15% of cosmetic procedure patients meet criteria for body dysmorphic disorder (BDD).^13^ The National Institute for Health and Care Excellence recommends that anyone with suspected BDD seeking cosmetic treatment be referred for mental health evaluation rather than simply going ahead with the procedure.^14^ Professional guidance in the United Kingdom (GMC [General Medical Council] and others) emphasizes that practitioners must consider patients' psychological needs and ensure realistic expectations before treatment.^15^

Despite its generally favorable safety, the rapid growth in BoNT use has outpaced the development of regulatory safeguards in the United Kingdom. An independent 2013 review of cosmetic interventions in the United Kingdom led by Sir Bruce Keogh concluded that “a person having a non-surgical cosmetic intervention has no more protection and redress than someone buying a ballpoint pen or a toothbrush,” highlighting the absence of effective regulation at that time.^16^ Unlike most medical treatments, cosmetic BoNT procedures in the United Kingdom could long be carried out by virtually any individual—including non-medical practitioners—provided a licensed prescriber supplied the product. This permissive environment, combined with aggressive commercial marketing, raised serious patient safety and ethical concerns.^15^ Key professional guidelines have since been introduced to encourage more ethical practice (eg, the GMC's 2016 guidance prohibits doctors from offering inappropriate incentives or performing treatments without assessing patients' psychological needs).^15^ For example, remote prescribing of BoNT is now explicitly banned by UK regulators.^15^ Recent legislation has also addressed 1 major gap by making it illegal to administer BoNT for cosmetic purposes to minors under 18 years of age (effective October 2021).^17^ However, beyond the under-18 age group, much of the cosmetic injectable industry in the United Kingdom remains less regulated than the medical community has long called for.

The United Kingdom presents a uniquely pertinent context for studying cosmetic BoNT use, despite soaring demand for esthetic injectables, the United Kingdom remains one of the only high-income countries where non-healthcare professionals can legally administer BoNT under minimal regulatory oversight. Recent government consultations and the anticipated implementation of a national licensing scheme reflect growing concern around patient safety, ethical standards, and practitioner accountability. Against this backdrop, we conducted a national observational survey with the aim of capturing the real-world experiences of cosmetic BoNT in the United Kingdom. This manuscript presents the survey findings, focusing on patient-reported outcomes, adverse events, injector qualifications, and regulatory awareness.

## METHODS

### Study Design

A cross-sectional observational survey was designed to gather data on individuals' experiences with cosmetic BoNT injections across Great Britain and Northern Ireland.^18^ The survey was developed collaboratively, via working groups, by academic researchers and clinicians, and was hosted online (using Jisc Online Surveys) to maximize accessibility. Participation was open to adults (≥18 years) residing in Great Britain or Northern Ireland who had ever undergone a BoNT treatment for esthetic purposes. Thus, the exclusion criteria were anyone under the age of 18 years, who had never undergone BoNT and resided outside Great Britain and Northern Ireland. Respondents were recruited via widespread dissemination on social media, cosmetic patient networks, and press releases. For example, the survey was advertised by The British Broadcasting Corporation Essex, circulated by numerous BoNT providers via their client lists, and embedded in the British College of Aesthetic Medicine newsletter, requesting that members circulate to their client lists, etc. The survey was anonymous and voluntary, with informed consent obtained at the start. Ethical approval for the study was granted by the Research Ethics Panel (Ethics ID: ETH2425-1930).

### Survey Instrument

The questionnaire comprised 34 items, combining multiple-choice and free-text responses, and was structured into sections: (1) Demographics—age, gender, and geographic region; (2) Experience and treatment details—number of sessions, the type of BoNT product received (eg, Botox [Allergan Aesthetics, an Abbvie Company, Irvine, CA], Dysport/Azzalure [Galderma, Fort Worth, TX], Bocouture/Xeomin [Merz Pharmaceuticals, Frankfurt, Germany]), the clinical setting where treatment occurred (eg, medical clinic, beauty salon), whether the prescriber was present at the time of consultation or injection, and whether pretreatment information on potential complications and consent was provided; (3) Satisfaction and adverse events—overall satisfaction with treatment results and any complications experienced (to capture psychological impacts, adverse event options such as anxiety, mood changes, and emotional distress were included, recognizing that such effects often accompany cosmetic treatment experiences); (4) Redress—if an adverse event occurred, what support was provided by the clinic and where the patient sought treatment for the adverse event. Patients were also asked whether their clinic or practitioner told them about the Yellow Card Reporting Scheme. Through the Yellow Card scheme, the UK Medicine and Healthcare Products Regulatory Agency collects and monitors information reported by the public on suspected safety concerns involving healthcare products, like side effects caused by a medicine, or adverse incidents involving medical devices; and (5) Regulation and knowledge—awareness of the practitioner's qualifications and regulatory status, and opinions on industry regulation.

For adverse events, respondents selected from a list of common BoNT acute and long-term side effects, including a “Never experience an acute complication” and “Never experience a long-term complication” option, and a check box available for other known adverse events. Long-term effects were defined as problems persisting beyond the immediate post-injection period, and multiple responses were permitted. The survey also probed as to whether respondents had been informed prior to treatment about potential complications, and whether formal informed consent was obtained.

Due to the retrospective, self-reported nature of the survey, specific dosage details (eg, units of toxin administered) were not collected, as most patients are unlikely to be informed of or recall this information accurately.

A pilot test involving 5 participants was conducted to ensure the questionnaire's clarity and appropriate length, following which no adjustments were necessary. The final survey was launched on December 12, 2024 and remained open for 4 months.

### Data Analysis

Survey responses were exported and analyzed using descriptive statistical methods. Categorical data (eg, proportions reporting each type of complication) are presented as absolute frequencies and percentages. No imputation was conducted for missing responses; participants who submitted incomplete surveys were included in the analysis for all items they did complete, and item-level completion rates are reported accordingly.

Free-text comments were subjected to thematic analysis to identify common patterns in respondent narratives, with particular attention paid to issues of regulatory awareness, perceived professional status of injectors, and the financial and social ramifications of outcomes. Detailed reporting of qualitative findings will be provided in a separate publication.

The present descriptive paper aims to describe the sample and the overall responses, highlighting the current state of BoNT practice in the United Kingdom in relation to the carefully selected measures. Future papers will investigate associations such as between BoNT administration type (eg, nurse vs doctor vs beautician) and risk of adverse events. Therefore, inferential statistics were not employed in the present work. Instead, principal outcome metrics, such as patient satisfaction rates, complication prevalence, and redress pathways, are reported and contextualized.

## RESULTS

A total of 919 participants completed the survey and were included in the present paper (Supplemental Table 1). However, as already noted, participants who submitted incomplete surveys were included in the analysis for all items they did complete. Thus, the total number of responses varies for each survey question; we highlight this throughout the results.

### Regulation, Satisfaction, and Complaint-Related Findings

Most respondents reported a high level of satisfaction with their treatment, with 89.8% (*n* = 818) expressing positive views out of 911 participants. Formal complaints were uncommon, filed by only 5.3% (*n* = 47) of 894 individuals. Awareness of existing regulations was limited, as just 35.4% (*n* = 317) of 896 respondents stated they were familiar with current policies. Regarding perceptions of regulatory stringency, 31.3% (*n* = 281) believed that regulation should be somewhat stricter, 57.8% (*n* = 519) supported significantly stricter oversight, and 10.9% (*n* = 98) felt that regulations should be relaxed. Notably, awareness of the national adverse event reporting system (“yellow card”) was also low, with only 26.5% (*n* = 233) of 880 participants aware of its existence. Results are presented in Supplemental Table 2.

### Mental Health Status of Respondents

**Figure 1.**
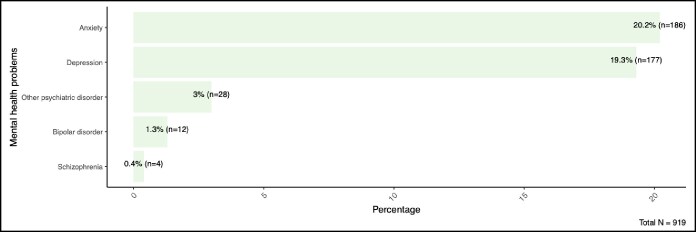
Frequency of mental health diagnoses among participants.

Of the 919 individuals surveyed, 20.2% (*n* = 186) reported having anxiety, and 19.3% (*n* = 177) reported having depression. In contrast, only 1.3% (*n* = 12) reported a diagnosis of bipolar disorder, 0.4% (*n* = 4) reported schizophrenia, and 3.0% (*n* = 28) indicated other psychiatric conditions (Figure 1).

### Physical Health Conditions

**Figure 2.**
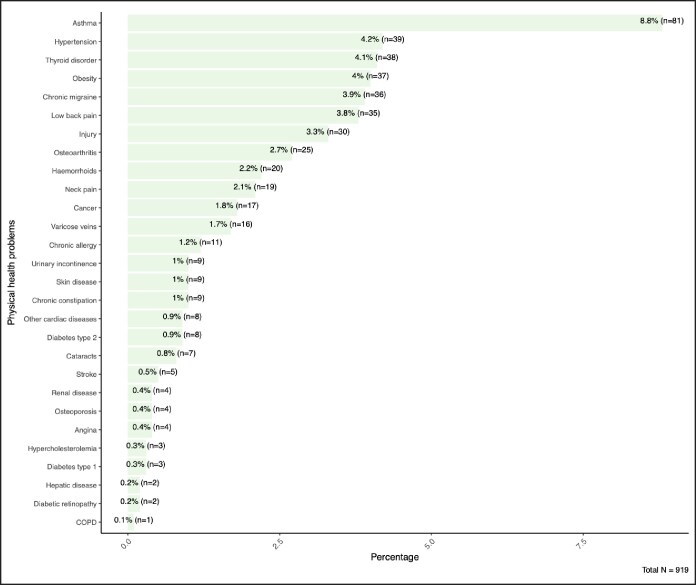
Frequency of physical health concerns among participants.

Among the 919 respondents, the most frequently reported existing physical health conditions were asthma (8.8%, *n* = 81), obesity (4.0%, *n* = 37), hypertension (4.2%, *n* = 39), chronic migraine (3.9%, *n* = 36), and low back pain (3.8%, *n* = 35). Other common complaints included injury-related issues (3.3%, *n* = 30), osteoarthritis (2.7%, *n* = 25), and neck pain (2.1%, *n* = 19). Conditions with lower prevalence included hemorrhoids (2.2%, *n* = 20), varicose veins (1.7%, *n* = 16), cancer (1.8%, *n* = 17), and thyroid disorders (4.1%, *n* = 38). Rare conditions (each affecting ∼1% of the sample) included chronic allergy (1.2%, *n* = 11), chronic constipation (1.0%, *n* = 9), chronic obstructive pulmonary disease (0.1%, *n* = 1), angina (0.4%, *n* = 4), stroke (0.5%, *n* = 5), skin disease (1.0%, *n* = 9), osteoporosis (0.4%, *n* = 4), renal disease (0.4%, *n* = 4), diabetic retinopathy (0.2%, *n* = 2), and urinary incontinence (1.0%, *n* = 9) (Figure 2).

### Sociodemographic Characteristics and Healthcare Access

Of the 918 participants, 90.8% (*n* = 834) identified as female and 9.2% (*n* = 84) as male. The majority were of White ethnicity (82.7%, *n* = 760), while 17.3% (*n* = 159) identified as non-White. Most respondents resided in England (92.8%, *n* = 844), followed by Scotland (3.2%, *n* = 29), Northern Ireland (2.2%, *n* = 20), and Wales (1.8%, *n* = 16). In terms of age, the most represented groups were 35 to 44 years (36.7%, *n* = 337), 25 to 34 years (29.0%, *n* = 266), and 45 to 54 years (17.8%, *n* = 163), with only 0.2% (*n* = 2) aged 75 or older. Income distribution was skewed toward higher earners, with 52.0% (*n* = 472) of respondents reporting annual incomes above £60,000. In contrast, only 3.2% (*n* = 29) earned less than £15,000, and 7.2% (*n* = 65) fell within the £15,000–25,000 range. Sociodemographic characteristics are presented in Supplemental Table 1.

### Healthcare Access and Additional Costs

Regarding healthcare access, 95.6% (*n* = 879) of the 919 respondents had not consulted their general practitioner (GP) about treatment complications, and 96.4% (*n* = 886) had not sought help from other healthcare providers. Additionally, 98.4% (*n* = 904) had not contacted The National Health Service (NHS) 111 (a free, non-emergency medical helpline and online service available 24/7 in England) for related issues. Concerning additional costs in relation to complications of BoNT treatment, among the 202 respondents to that item, 73.3% (*n* = 148) reported no extra payments, 21.8% (*n* = 44) had paid privately, and 5.0% (*n* = 10) incurred costs through the NHS. Results are shown in Supplemental Table 2.

### Consent and Information Disclosure

Among the 911 participants who responded to the consent-related question, 8.5% (*n* = 77) reported that they were not provided with a written consent form prior to their treatment, while 91.5% (*n* = 834) indicated that they received one. Regarding the information provided about potential risks and complications, 10.9% (*n* = 100) of respondents stated they were not informed about risks, and 17.8% (*n* = 163) reported not being informed about possible complications. See Supplemental Table 2 for results.

### Acute Complications Following Treatment

**Figure 3.**
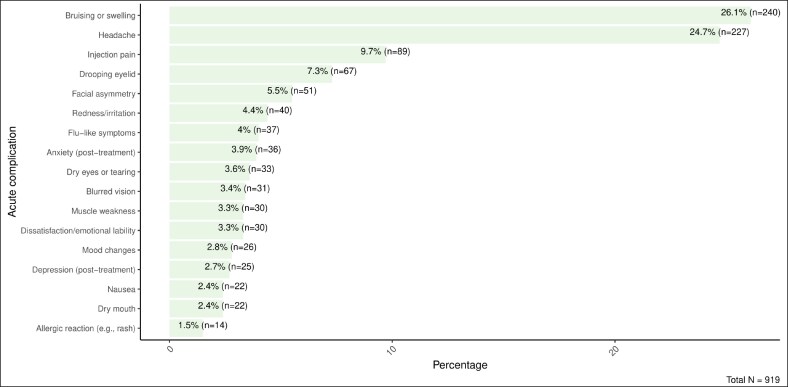
Frequency of acute complications secondary to botulinum toxin treatment among participants.

Among the 919 respondents, the most commonly reported acute complication was bruising or swelling (26.1%, *n* = 240), followed by headache (24.7%, *n* = 227) and injection site pain (9.7%, *n* = 89). Other frequently cited events included drooping eyelid (7.3%, *n* = 67), facial asymmetry (5.5%, *n* = 51), redness or irritation (4.4%, *n* = 40), flu-like symptoms (4.0%, *n* = 37), and dry eyes or tearing (3.6%, *n* = 33). Less common complications were anxiety (3.9%, *n* = 36), blurred vision (3.4%, *n* = 31), muscle weakness (3.3%, *n* = 30), emotional lability or dissatisfaction (3.3%, *n* = 30), mood changes (2.8%, *n* = 26), depression (2.7%, *n* = 25), dry mouth (2.4%, *n* = 22), nausea (2.4%, *n* = 22), itching (1.5%, *n* = 14), and allergic reactions such as rash (1.5%, *n* = 14) (Figure 3).

### Long-Term Complications

**Figure 4.**
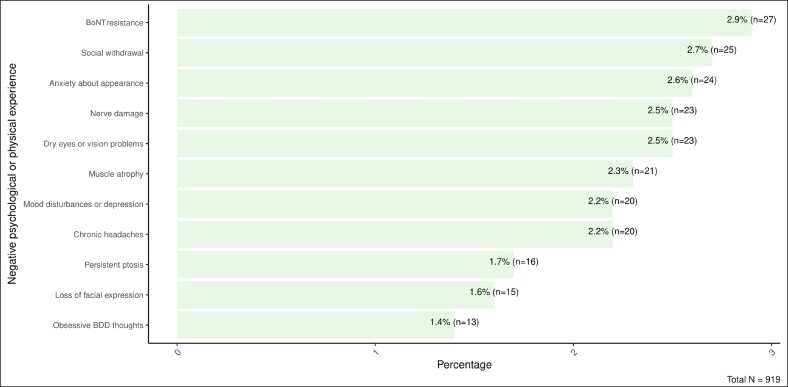
Frequency of negative psychological or physical experiences secondary to botulinum toxin treatment among participants.

Among the 919 individuals surveyed, long-term complications were relatively rare (Figure 4). The most commonly reported were BoNT resistance (2.9%, *n* = 27), social withdrawal (2.7%, *n* = 25), nerve damage (2.5%, *n* = 23), and dry eyes or vision problems (2.5%, *n* = 23). Other outcomes included muscle atrophy (2.3%, *n* = 21), chronic headaches (2.2%, *n* = 20), mood disturbances or depression (2.2%, *n* = 20), and anxiety about appearance (2.6%, *n* = 24). Less frequently reported complications were persistent ptosis (1.7%, *n* = 16), loss of facial expression (1.6%, *n* = 15), and obsessive thoughts related to BDD (1.4%, *n* = 13).

### Treatment Characteristics, Clinical Context, and Patient Motivations and Experiences

Most participants had received BoNT treatment more than 5 times (54.4%, *n* = 497), while 33.4% (*n* = 305) had undergone it 2 to 5 times, and 12.2% (*n* = 111) only once (Supplemental Table 2, *n* = 826). Regarding treatment frequency, the most common interval was every 4 to 5 months (41.9%, *n* = 346), followed by every 6 to 7 months (22.5%, *n* = 186). Monthly applications were rare (0.1%, *n* = 1) (Supplemental Table 2, *n* = 913). In terms of product type, the most commonly used was Botox (39.4%, *n* = 352), followed by Dysport/Azzalure (20.5%, *n* = 183) and Bocouture/Xeomin (10.4%, *n* = 93), while 24.7% (*n* = 221) of participants did not know which product had been used (Supplemental Table 2, *n* = 894).

**Figure 5.**
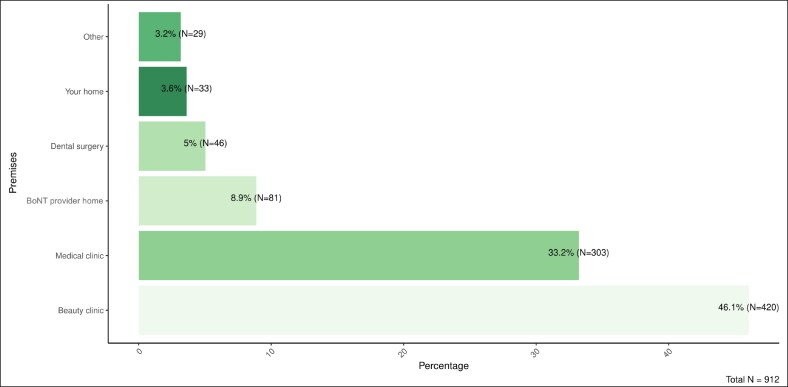
Type of premises where the botulinum toxin treatment was received.

**Figure 6.**
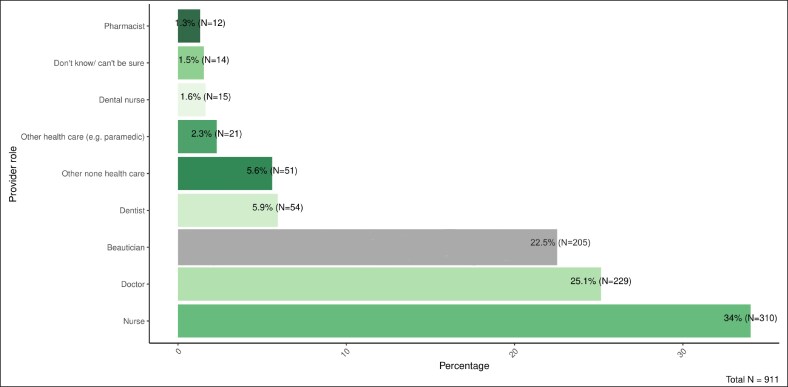
Qualifications of the provider delivering the botulinum toxin treatment.

Treatment was most commonly received in beauty clinics (46.1%, *n* = 420), followed by medical clinics (33.2%, *n* = 303), dental surgeries (5.0%, *n* = 46), and home environments, either the provider's (8.9%, *n* = 81) or the participant's (3.2%, *n* = 29) (Figure 5, *n* = 912). The most frequent provider roles were nurses (34.0%; *n* = 310), doctors (25.1%, *n* = 229), and beauticians (22.5%, *n* = 205) (Figure 6, *n* = 911). Only 42.4% (*n* = 199) of participants reported that a prescriber was present during the consultation, 40.3% (*n* = 189) indicated no prescriber was present, and 17.3% (*n* = 81) did not know (Supplemental Table 2, *n* = 469). Regarding the treatment administration, 61.2% (*n* = 248) confirmed that the prescriber was not present at the moment of injection (Supplemental Table 2, *n* = 405). Concerning qualifications, 65.8% (*n* = 601) believed the person administering the treatment held appropriate prescribing credentials, while 28.4% (*n* = 260) did not, and 5.8% (*n* = 53) were unsure (Supplemental Table 2, *n* = 914).

Despite the high treatment uptake, only 26.3% (*n* = 85) of participants reported receiving any form of support from the clinic after the procedure, while 73.7% (*n* = 238) did not (Supplemental Table 2, *n* = 323). Additionally, just 28.4% (*n* = 74) recalled receiving advice on how to manage potential adverse effects, whereas 71.6% (*n* = 187) did not receive such guidance (Supplemental Table 2, *n* = 261).

In terms of motivations, 80.5% (*n* = 740) reported anti-ageing reasons, and 61.3% (*n* = 564) identified cosmetic preference. Only 11.0% (*n* = 101) cited medical reasons, and 3.2% (*n* = 29) acknowledged peer influence (Supplemental Table 2, *n* = 919). Regarding satisfaction, 51.2% (*n* = 459) felt the treatment met their expectations, 40.6% (*n* = 364) reported that it exceeded them, and only 8.1% (*n* = 73) stated it fell short (Supplemental Table 2, *n* = 896).

## DISCUSSION

This national survey represents the most comprehensive analysis to date of patient-reported experiences and outcomes associated with cosmetic BoNT use in the United Kingdom. The data affirm that when administered appropriately, BoNT type A (BoNT-A) is a well-tolerated and effective intervention for facial esthetic enhancement.^3,18^ Approximately 90% of surveyed individuals reported satisfaction with treatment outcomes, and the majority did not experience adverse effects, findings that are consistent with clinical trial data and systematic reviews characterizing BoNT-A as a low-risk, high-satisfaction cosmetic treatment.^3,4,6-9^ The acute complication rate observed in this survey (∼18%) is comparable to the 16% pooled incidence reported in a recent meta-analysis, suggesting that real-world outcomes generally mirror those achieved in structured clinical environments.^11^

However, this positive safety and satisfaction profile coexists with critical concerns highlighted by the survey. Leading among these are the under-recognition of complications, inconsistent regulation and training standards, variable qualifications among providers, gaps in informed consent, psychosocial implications of esthetic treatment, and burden-shifting onto public healthcare services.

### Complication Prevalence and Underreporting

Although serious adverse events were infrequent in our sample, the survey confirms that complications are neither rare nor uniformly reported. Yet, national pharmacovigilance data from the UK's Medicines and Healthcare products Regulatory Agency (MHRA) suggest far fewer events are formally documented. From 1991 to 2020, only 188 adverse events associated with cosmetic BoNT were recorded, an average of fewer than seven cases per year, despite an estimated 900,000 procedures being performed annually in the United Kingdom.^2,12^ This discrepancy suggests a substantial underreporting gap.

The reasons for this underreporting are likely multifactorial. Patients may be unaware of how or where to report complications, highlighted in the present findings by being unaware of the Yellow Card reporting scheme, may interpret minor side effects as expected outcomes, or may be reluctant to disclose dissatisfaction due to embarrassment or perceived stigma. Similarly, non-healthcare practitioners, who represent a significant proportion of the injector workforce, may lack formal reporting obligations.

To address this, the establishment of a standardized, mandatory reporting mechanism, either within the MHRA's Yellow Card scheme or through a dedicated esthetic treatment registry, is advisable. Moreover, practitioners, regardless of their profession, should make all patients aware of the MHRA's Yellow Card scheme and encourage them to report any adverse events that they may experience.

### Regulatory Gaps and Evolving Oversight

The findings of this survey reinforce longstanding concerns about the inadequacy of regulatory oversight in the UK cosmetic injectables sector. For example, in 2017, the Nuffield Council on Bioethics characterized the UK's regulatory regime for esthetic procedures as “completely inadequate.”^19^ The Council highlighted gaps in public understanding, inconsistent training among providers, and limited avenues for patient redress.^20^ Our present findings suggest that many of these structural shortcomings remain unaddressed. Patients continue to receive treatments in unregulated settings from providers with variable or unclear qualifications, and formal complaint pathways remain underutilized.

Importantly, the UK regulatory environment remains heterogeneous. England is preparing to launch its national licensing scheme, while Wales has introduced licensing for certain procedures, initially excluding injectables such as BoNT, a decision that has drawn criticism from public health advocates. In contrast, Scotland has required independent esthetic clinics run by healthcare professionals to register with Healthcare Improvement Scotland since 2016. Northern Ireland retains its own regulatory mechanisms, though harmonization with broader UK reforms is anticipated.

### Practitioner Qualifications and Ethical Standards

One of the most striking findings of this survey is the pronounced variability in practitioner backgrounds among those performing cosmetic BoNT injections in the United Kingdom. Respondents reported receiving treatment from a range of providers, including medical doctors, esthetic nurses, pharmacists, dentists, and beauticians. This professional diversity reflects the absence of mandatory credentialing or statutory licensing for esthetic practitioners in the United Kingdom, which currently permits both medically and non-medically trained individuals to offer injectable treatments under minimal to zero oversight.

Our findings mirror national data on provider composition. A recent audit of over 4400 individuals offering cosmetic injectables in the United Kingdom found that only 32% were doctors, 13% were nurses, and ∼12% were non-medical estheticians.^21^ The remaining providers included pharmacists, dentists, and others operating outside traditional medical regulation. Compounding this fragmented landscape is the existence of more than 20 professional associations or self-designated accrediting bodies for esthetic practice, many of which impose minimal entry requirements. Fewer than 15% require an in-person skills assessment as part of their credentialing process.^22^

While many medical professionals in esthetic medicine follow robust ethical frameworks, the permissive regulatory environment has potentially enabled the persistence of unsafe or unethical practices. The media and professional bodies have reported instances of unregulated or undertrained practitioners, as well as concerning practices by registered clinicians, including off-label or remote prescribing without adequate clinical assessment, and superficial or omitted consent discussions. Such practices contravene ethical guidelines established by the GMC and the Royal College of Surgeons, both of which emphasize that commercial incentives must never override clinical judgment or patient welfare.^15^

In our survey, potential breaches of ethical best practice were reported by participants. A notable proportion of respondents reported not receiving a written consent form (8.5%), not being informed of risks (10.9%), or lacking guidance on managing complications (17.8%), all fundamental ethical obligations under the GMC and other regulatory guidance. All practitioners must ensure that written informed consent is received prior to BoNT treatment, patients are informed of the risks relating to the treatment, and clear guidance is provided to the patient in relation to managing complications. Indeed, non-healthcare professionals are not bound by statutory codes of conduct or professional disciplinary mechanisms. Unlike regulated clinicians, such individuals are under no legal obligation to uphold ethical standards, nor do they face fitness-to-practice investigations in the event of misconduct.

In response to such concerns, regulatory authorities have introduced several targeted reforms. In 2016, the GMC issued formal guidance requiring that all patients receiving BoNT undergo a face-to-face consultation with the prescribing clinician, thereby prohibiting the previously widespread practice of remote prescribing. This has recently been mirrored by the Nursing and Midwifery Council (NMC) from only April 2025. In parallel, the UK Committee of Advertising Practice, working with the Advertising Standards Authority, has banned all direct-to-consumer advertising of prescription-only medicines, including BoNT.

Despite these measures, our data suggest that substantial numbers of patients continue to undergo treatment without a full understanding of risks or recourse. This highlights a broader ethical deficit that transcends individual bad actors: the failure to embed comprehensive, patient-centered communication within routine esthetic practice. Addressing this will likely require not only enhanced practitioner education and clearer patient-facing materials but also regulatory audit mechanisms to ensure adherence to ethical and consent standards across the industry.

### Psychological Considerations in Cosmetic BoNT Use

In our survey, although overall satisfaction rates were high, a proportion of respondents reported adverse psychological outcomes. These included episodes of acute anxiety or mood disturbance following treatment, as well as more enduring emotional sequelae. These findings suggest that elective esthetic procedures may, in some individuals, exacerbate underlying mental health vulnerabilities rather than ameliorate them. Despite these potential risks, psychological screening is not yet a routine part of practice in the UK cosmetic sector.^14^

### Public Health Implications and the Burden on the NHS

Although cosmetic procedures are generally framed as elective and privately funded services, the survey findings demonstrate a clear interface with public health systems, particularly when complications occur. Among respondents who experienced adverse outcomes following cosmetic BoNT injections, more than one-third sought assistance from the NHS.

Moreover, the psychological sequelae of cosmetic complications, particularly facial disfigurement, persistent asymmetry, or treatment failure, may lead to repeated GP visits, anxiety management, or referrals to mental health services. These indirect consequences, though harder to quantify, represent additional and potentially long-term demands on NHS capacity.

### Limitations

Several limitations of this study warrant consideration. As a voluntary, self-selected survey relying on patient recall, there is a potential risk of self-selection bias: individuals who experienced adverse outcomes may be more likely to participate than those with routine or uneventful treatments. Additionally, the absence of clinical validation means that transient or expected post-injection effects may have been misclassified as complications. For example, lay respondents may conflate eyebrow descent, asymmetry, or periocular heaviness with true clinical ptosis. Moreover, all self-reported surveys are subject to recall bias.

Another limitation is the lack of temporal resolution: the survey did not collect the specific dates of respondents' most recent treatments. Some individuals may have reported experiences from several years ago, before recent regulatory changes (such as the 2021 ban on under-18 cosmetic treatments or the 2022 licensing framework announcement). This may have influenced responses related to informed consent, prescriber involvement, and aftercare, particularly among early adopters of esthetic procedures. Furthermore, the survey did not collect data on the dosage (in units) of BoNT administered, which may limit direct comparisons with clinical trial data. Future studies should aim to integrate both self-reported outcomes and practitioner-reported dosage data for more comprehensive safety profiling. Finally, owing to the widespread dissemination of the survey, it was not possible to calculate the non-response rate.

Despite these limitations, the study's strengths, most notably its large sample size, geographic reach, and breadth of questions, support the validity of the findings.

### Implications for Practice, Policy, and Public Health

The findings of this national survey yield several important implications for clinical practice, regulatory policy, and broader public health strategy. First, the overwhelmingly high rates of patient satisfaction and the low incidence of serious complications are reassuring and should be communicated clearly to the public. Public education campaigns could help clarify expectations, differentiate normal post-treatment effects (eg, mild bruising or transient asymmetry) from true complications, and thereby reduce unnecessary concern or dissatisfaction.

Second, the survey highlights gaps in informed consent, aftercare, and practitioner qualification that require urgent attention. Regulatory authorities and professional training programs should prioritize not only technical competency in injection technique but also ethical conduct, communication skills, and patient-centered care. Practitioners must be trained to deliver comprehensive pretreatment counseling, set realistic expectations, screen for psychosocial risk factors, and provide structured follow-up pathways. Ethical safeguards must be universal. In our survey, nearly 1 in 10 patients did not sign a consent form, moreover ∼1 in 10 were not informed of risks, and nearly 1 in 5 were not told how to manage complications, clear breaches of informed consent. To protect patients, all esthetic injectors must be registered with a statutory authority and held to enforceable ethical standards.

Third, the findings provide a strong public mandate for regulatory reform. Both patient support for tighter oversight and the lived experiences of those harmed by inadequate practice lend weight to ongoing policy efforts. As England prepares to implement a statutory licensing scheme for non-surgical cosmetic procedures, the design and enforcement of that scheme will be critical. Minimum educational standards, compulsory adherence to an ethical code, and clear mechanisms for investigation and sanction must be central components. Collaboration with professional associations, including those representing nurses, pharmacists, and allied health providers, will be essential to achieve a balance between safety and accessibility.

Fourth, these findings further highlight the imperative for regulatory authorities and insurers to mandate consistent consumer-facing safeguards. The fact that 24.7% of patients did not know which BoNT product they received, and 18% were not told how to respond to complications, points to systemic failings in product transparency and patient information.

Fifth, the survey's insights into mental health outcomes and NHS utilization underscore the need to more closely integrate esthetic medicine into the broader healthcare ecosystem.

Finally, the data expose a critical mismatch between the scale of cosmetic BoNT use in the United Kingdom and the current regulatory architecture designed to govern it. While the MHRA regulates BoNT as a prescription-only medicine, and professional guidance from the GMC, NMC, and General Pharmaceutical Council prohibits remote prescribing and mandates informed consent, these safeguards are not legally binding across all providers, nor uniformly enforced.

In summary, cosmetic BoNT injections are now a common feature of the UK healthcare landscape, with the potential for both meaningful benefit and avoidable harm. A regulatory and professional infrastructure that reflects this duality, one that elevates standards, enhances patient protection, and integrates with public health priorities, is both timely and necessary. Our findings provide empirical support and direction for those reforms.

## CONCLUSIONS

The growing use of BoNT for cosmetic purposes in the United Kingdom exemplifies a modern healthcare paradox: a medically regulated drug widely used in a largely unregulated esthetic marketplace. This national survey, the largest of its kind in the United Kingdom, confirms that cosmetic BoNT can deliver high satisfaction and a favorable safety profile when administered appropriately. However, the findings also reveal significant vulnerabilities, chiefly, inconsistent practitioner qualifications, gaps in informed consent, insufficient complication support, and a lack of robust regulatory infrastructure.

## Supplemental Material

This article contains supplemental material located online at https://doi.org/10.1093/asjof/ojaf115.

## Supplementary Material

ojaf115_Supplementary_Data
