# Supplementary material for: Findings From the Great British and Northern Ireland Botulinum Toxin Survey: Treatment Outcomes, Patient Experience, and Regulations From a Cross-Sectional Survey
Source: Aesthet Surg J Open Forum. 2025 Sep 16;7:ojaf115. doi: 10.1093/asjof/ojaf115 (PMC12578598; doi:10.1093/asjof/ojaf115)
Supplement: ojaf115_Supplementary_Data [file ojaf115_supplementary_data.zip › Supplementary Table 1_ .docx]

Table 1. Participants characteristics

|  | N (%) |
| --- | --- |
| **Age** |  |
| 18 - 24 | 65 (7.1) |
| 25 - 34 | 266 (29) |
| 35 - 44 | 337 (36.7) |
| 45 - 54 | 163 (17.8) |
| 55 - 64 | 69 (7.5) |
| 65 - 74 | 16 (1.7) |
| ≥75 | 2 (0.2) |
| **Gender** |  |
| Female | 834 (90.8) |
| Male | 84 (9.2) |
| **Ethnicity** |  |
| White | 760 (82.7) |
| Not White | 159 (17.3) |
| **Income** |  |
| < £15,000 | 29 (3.2) |
| £15,000 – £24,999 | 65 (7.2) |
| £25,000 – £39,999 | 149 (16.4) |
| £40,000 – £59,999 | 193 (21.3) |
| ≥ £60,000 | 472 (52) |
| **Location** |  |
| England | 844 (92.8) |
| Scotland | 29 (3.2) |
| Northern Ireland | 20 (2.2) |
| Wales | 16 (1.8) |
