# Supplementary material for: Findings From the Great British and Northern Ireland Botulinum Toxin Survey: Treatment Outcomes, Patient Experience, and Regulations From a Cross-Sectional Survey
Source: Aesthet Surg J Open Forum. 2025 Sep 16;7:ojaf115. doi: 10.1093/asjof/ojaf115 (PMC12578598; doi:10.1093/asjof/ojaf115)
Supplement: ojaf115_Supplementary_Data [file ojaf115_supplementary_data.zip › Supplementary Table 2_ .docx]

Table 2 Participant Experiences and Perceptions of Cosmetic Botulinum Toxin Treatment

|  | N (%) |
| --- | --- |
| **Satisfaction** |  |
| Yes | 818 (89.8) |
| No | 93 (10.2) |
| **Filed complaint** |  |
| Yes | 47 (5.3) |
| No | 847 (94.7) |
| **Regulation awareness** |  |
| Yes | 317 (35.4) |
| No | 579 (64.6) |
| **Stricter regulation opinion** |  |
| Yes, significantly stricter | 519 (57.8) |
| Yes, somewhat stricter | 281 (31.3) |
| No, regulations should be relaxed | 98 (10.9) |
| **Yellow card awareness** |  |
| Yes | 233 (26.5) |
| No | 647 (73.5) |
| **Additional costs** |  |
| Yes, through NHS | 10 (5) |
| Yes, paid privately | 44 (21.8) |
| No additional cost | 148 (73.3) |
| **Consulted GP about treatment complications** |  |
| Yes | 40 (4.4) |
| No | 879 (95.6) |
| **Consulted other healthcare provider about treatment complications** |  |
| Yes | 33 (3.6) |
| No | 886 (96.4) |
| **Consulted service 111 about treatment complications** |  |
| Yes | 15 (1.6) |
| No | 904 (98.4) |
| **Signed treatment consent form** |  |
| Yes | 834 (91.5) |
| No | 77 (8.5) |
| **Informed about potential complications** |  |
| Yes | 753 (82.2) |
| No | 163 (17.8) |
| **Informed about potential risk** |  |
| Yes | 815 (89.1) |
| No | 100 (10.9) |
| **Total number of treatments** |  |
| 1 | 111 (12.2) |
| 2-3 | 134 (14.7) |
| 4-5 | 171 (18.7) |
| >5 | 497 (54.4) |
| **Treatment frequency** |  |
| Monthly | 1 (0.1) |
| Every two to three months | 91 (11) |
| Every four to five months | 346 (41.9) |
| Every six to seven months | 186 (22.5) |
| Every seven to eight months | 53 (6.4) |
| Every nine to ten months | 48 (5.8) |
| Every eleven to twelve months | 32 (3.9) |
| Fewer than once a year | 69 (8.4) |
| **Botulinum Toxin treatments product type** |  |
| Botox / Allergan | 352 (39.4) |
| Dysport / Azzalure | 183 (20.5) |
| Bocouture / Xeomin | 93 (10.4) |
| Nuceiva / Jeuveau | 38 (4.3) |
| Letybo | 7 (0.8) |
| Do not know | 221 (24.7) |
| **Consultation prescriber present** |  |
| Yes | 189 (40.3) |
| No | 199 (42.4) |
| Do not know | 81 (17.3) |
| **Treatment prescriber present** |  |
| Yes | 102 (25.2) |
| No | 248 (61.2) |
| Do not know | 55 (13.6) |
| **Prescriber qualification** |  |
| Yes | 601 (65.8) |
| No | 260 (28.4) |
| Do not know | 53 (5.8) |
| **Clinic adverse effects advice** |  |
| Yes | 74 (28.4) |
| No | 187 (71.6) |
| **Clinic support** |  |
| Yes | 238 (73.7) |
| No | 85 (26.3) |
| **Treatment reason** |  |
| Anti-ageing | 740 (80.5) |
| Cosmetic preference | 356 (38.7) |
| Medical reasons | 101 (11) |
| Peer influences | 29 (3.2) |
| **Treatment expectations** |  |
| Met expectations | 459 (51.2) |
| Exceeded expectations | 364 (40.6) |
| Did not meet expectations | 73 (8.1) |
